# Death from Swallowing Two Ounces of Chloroform

**Published:** 1867-10

**Authors:** 


### Death from Swallowing Two Ounces of Chloroform.

The following case, recorded by Dr. D. W. Stormont, of Topeka, Kansas, (*Leavenworth Medical Herald*, July, 1867,) is particularly interesting from the short time which intervened between taking the chloroform and death:

A healthy man, twenty-six years of age, for the purpose of self-destruction, at ten o'clock swallowed, in the presence of his obdurate sweetheart and a female friend, two ounces of undiluted chloroform. He then composedly laid down on a bed, as he said, to die. "In three minutes (estimated time) he could with difficulty be aroused from the stupor into which he was rapidly sinking; could not speak, but indicated that he had severe pain in the stomach. In five minutes he was entirely unconscious, lying still, breathing stertorously. He died in just one hour after taking the draught. Medical assistance, from some cause, did not arrive until a few minutes before he died, and nothing was done to counteract the effects of the poison."

*Post-mortem.*—"The surface was livid; the face, neck, chest, and nails very much so. Bloody froth was issuing from the mouth and nostrils. On opening the chest, both lungs were found to be dark externally, and fully distended. They were uniformly congested with dark, liquid blood, and the posterior portions were perfectly engorged with it. Both sides of the heart were nearly full of black, uncoagulated blood. The liver and spleen both normal externally, but somewhat softened, and filled with dark, liquid blood. The œsophagus was congested. The stomach, at the cardiac end, and along the greater curvature, and half-way up each side, was discolored externally, dotted over with echymosed looking patches, giving it a mottled appearance. It contained two or three ounces of a light-colored liquid, which had a slight odor of chloroform. At the cardiac end, internally, and along the bottom nearly to the pyloric end, the mucous membrane was of a dark-red color, softened, and easily peeled off with the thumb-nail. Up the sides it was of a brighter red, speckled appearance, and not softened. The intestines were healthy. Circumstances prevented us from extending the examination, which is to be regretted."—*Am. Journal Medical Sciences.*

